# Supplementary material for: Impact of Biological Agents on Postsurgical Complications in Inflammatory Bowel Disease: A Multicentre Study of Geteccu
Source: J Clin Med. 2021 Sep 26;10(19):4402. doi: 10.3390/jcm10194402 (PMC8509475; doi:10.3390/jcm10194402)
Supplement: Supplementary file 1 [file jcm-10-04402-s001.zip › jcm-1392810-supplementary.pdf]

Supplementary Material

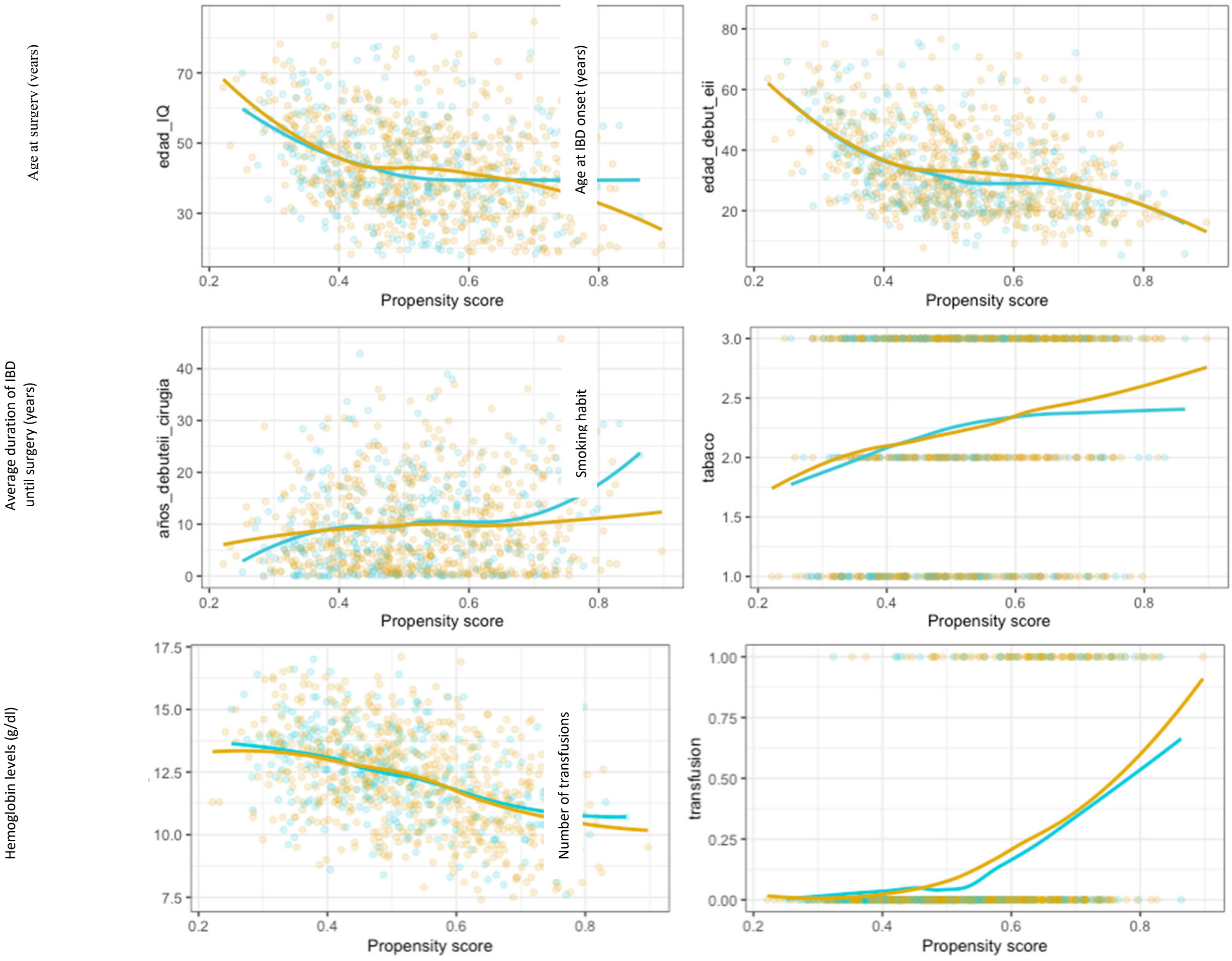

Figure S1. Relationship between means and propensity scores for different clinical variables. The blue line represents the non-exposed cohort and the yellow one the exposed cohort.
